# Supplementary material for: A generalized higher-order correlation analysis framework for multi-omics network inference
Source: PLoS Comput Biol. 2025 Apr 14;21(4):e1011842. doi: 10.1371/journal.pcbi.1011842 (PMC11996223; doi:10.1371/journal.pcbi.1011842)
Supplement: Attachment — Submitted filename: PLOSCompBIO- revision-KK.docx [file pcbi.1011842.s002.docx]

Dear Editor,

Thank you so much for the quality assessment on our manuscript “A Generalized Higher-order Correlation Analysis Framework for Multi-Omics Network Inference”. We have carefully revised it based on your feedback and have made corresponding changes. Below, we respond to each point:

**Comments to the Authors:
Please note here if the review is uploaded as an attachment.**

Reviewer #1: In “A Generalized Higher-order Correlation Analysis Framework for Multi-Omics Network Inference”, the authors present a new method for constructing small networks tied to defined phenotypes (or other descriptive data) that represent multi-view high dimensional data.

It’s an important avenue for research; multi-view data integration is extremely difficult, in many ways, but also rapidly becoming more common and necessary for analysis. Tools will therefore be very welcome.

The authors provide two worked examples, one being TCGA breast cancer data and the other being a COPD data set. In both cases, the algorithm produces fairly small networks, composed of connected node-features representing the multi-view, and show the networks are more closely related to the selected phenotypes compared to a competitive algorithm.

Thoughts:

(A) Multi-view data integration is especially difficult in biomedical situations where the data depends on tissue biopsies (or other tissue samples). The wet bench assays that produce the data, by nature destroy the samples, which make true multi-view data nearly impossible. There are some exceptions with more recent assay types such as CITE-sea and image based spatial sequencing data, but they are not completely mature data types.

In many cases, tissue structure related to disease tends to be high heterogeneous in its structure — that can relate to disease progression or subtype— and by splitting samples or using unrelated samples as multi-views, there will naturally be some disagreement or noise, discord, between the views because they are not views of the same bulk collection of cells. I expect this application is more difficult compared to other applications where there really is different views of exactly the same thing (like scans of the sun).

**Then what I wonder about is the stability of the result. Is the resulting network robust? I think this could be explored by performing a leave-one-out procedure, where you drop one sample, run the pipeline, and compare the collection of resulting networks. Is there a large stable core of connected nodes? Do the weak nodes fall off more often than strong nodes? Maybe not. It would be surprising, but neat, if weak nodes turned out to be sample with variability in the sample set.**

*We appreciate the reviewer’s comment on the stability of the network. The network stability is already built into SGTCCA-Net with the biased-subsampling algorithm where we subsample for multiple times. While the leave-one-out approach can be extremely intensive for SGTCCA-Net on COPDGene data because of the sample size, we managed to run it on the smaller TCGA breast cancer data.*

***Section 0.14:*** *We also tested the stability of the network by implementing a leave-one-out approach. During every iteration, we took out one subject and ran SGTCCA-Net on the remaining 104 subjects, generating 105 different multi-omics networks. After running SGTCCA-Net, we found a stable consistent core of the network: 79 molecular features appeared in every network, and 136 molecular features appeared at least 100 times out of 105 networks. All of these features are part of the original network based on the all subjects. Based on these results, we can conclude that the network's core structure is highly stable, demonstrating the reliability of SGTCCA-Net in identifying significant molecular features across different subsets of the data. Furthermore, of these 136 molecular features, only one is not significantly associated with tumor purity, while among the remaining 164 molecular features in the original network, 54 are not significantly associated with tumor purity (FDR > 0.05). This indicates that the strongest nodes obtained from SGTCCA-Net are less likely to be removed than the weaker nodes. These findings demonstrate the robustness of SGTCCA-Net in capturing key molecular features relevant to tumor purity, enhancing its potential utility in multi-omics research.*

**(B) In equation 1. It would be good to start out slow and bring the readers up to speed. So, right away defining things like w and X. What are their dimensions? What is the dimension of the output? Also you have parameters on the left hand side (w_1, w_2) and it seems like it should be more like F(w_1, w_2)=… ? Same for equation 3, there are parameters on the left-hand side. Generally, just read through all the written equations one more time, checking for little details.**

*We appreciate the reviewer’s suggestion on bringing readers up to speed with a more concrete introduction and have adjusted accordingly. Regarding the problem from Equation 1 reviewer points out, this is because we’re using argmax argument to formulate the problem. Argmax means “the input points at which a function output value is maximized”. However, we realize that definition inconsistencies may cause confusion. Therefore, we adjusted Equation 1 so that it is in the form of max rather than argmax.*

*We also proofread all the equations throughout the manuscript and make sure that they’re not difficult to understand. For example, we provided an updated definition of i-mode product that is easier for reader to understand:*

**(C) At line 80, you bring up the fact that higher order correlations may not be composed of multiple pair wise, or lower order, correlations. This could be expanded on with a concrete example, because this comes up later, and by explaining it here, the first time the reader sees it, then when it’s encountered later on, the reader already has a good grasp on this. It may not be intuitive. Plus, it goes in the other direction also, lower order correlations are not necessarily always contained in the higher order correlations. This is important as it comes up in the simulation results. The real difference comes from using high and low order correlations in parallel.**

*We appreciate reviewer’s suggestions on providing concrete examples of why a higher-order correlation does not always imply a pairwise or lower-order correlation, and we added the following paragraphs to support our argument:*

***Section 0.6.1:*** *……As mentioned earlier, the higher-order relationship may not imply multiple lower-order or pairwise relationships, and vice versa. This can be conceptually validated by a concrete example: suppose there are 4 different feature vectors z1, z2, z3, z4 ∈ R^N^ ×1. According to Equation 5, the 4-way higher-order covariance value between them can be reformulated as:*

*ρ(z1, z2, z3, z4) = | 1n (z1,2,3 z4)T 1|, (6)*

*where z1,2,3 = z1z2z3. In one particular scenario, a strong 4-way correlation between z1, z2, z3, z4 can be constructed by letting z1, z2, z3 be independent of each other, and z4 be z3 plus a small random noise. In this case, the 4-way correlation between z1, z2, z3, z4 does not imply the 3-way correlation between z1, z2, z3; In another particular scenario, a strong 3-way correlation between z1, z2, z3 can be constructed by letting z1, z2, z3 be strongly correlated with each other, but if z4 is independent of z1,2,3, then there does not exist the 4-way correlation between z1, z2, z3, z4……*

**(D) In section 0.6.1, the difficulty in interpretation of directionality of higher order correlations is briefly discussed. It’s an unfortunate fact, because from a modeling standpoint, that directionality is what we want. When gene x goes up, what happens to protein y and metabolite z?

So then, is the problem addressed by making the correlations absolute value (eq 4.)? Maybe I don’t see where this is directly addressed.**

*We appreciate the suggestions and have included one paragraph in the discussion section explaining that directionality is the drawback of the higher-order correlation. However, post-hoc analysis can be performed afterwards to can be used to interpret directionality between molecular features:*

***Discussion:*** *“One potential drawback of higher-order correlation is that it measures the simultaneous relationship among multiple features, making it difficult to determine the directionality of effects through mathematical formulation alone. However, a post-hoc analysis can be conducted on the higher-order correlation results to examine the direction of effect for each pair of features. For example, after identifying the 4-way correlation between a gene, a protein, a metabolite, and a phenotype, a post-hoc analysis can be performed to look at the pairwise Pearson's correlation between each pair of features, such as the gene and protein. This approach allows researchers to investigate the directionality of relationships based on the sign of the Pearson’s correlation between individual pairs of features, providing a clearer understanding of the underlying biological interactions.”*

*Additionally, in our experiments, we have included paragraphs that examined the pairwise association between molecular features using Pearson’s correlation. For example, in TCGA breast cancer experiment, we provided the following example:*

***Section 0.14:*** *“We calculate the PageRank score for the SGTCCA-Net subnetwork and present the top 5 nodes for each molecular profile in Table 3. We observed that all of the molecular features in the table are significantly correlated with tumor purity. ACVRL1 and KAT2A are the only two nodes with low correlation to tumor purity (FDR > 0.001). To check whether these nodes contribute to the network, we examine the correlation between these two nodes and other molecular features of the subnetwork by looking at their top 5 connections (based on Pearson’s correlation) to other molecular features. We observed that ACVRL1 is moderately correlated with PCNA (ρbetween−omics = 0.474), which is highly correlated with tumor purity (ρomics−pheno = −0.564) compared to other network proteins (rank 1/175 among all proteins) and KAT2A (ρbetween−omics = 0.476), which is highly correlated with tumor purity (ρomics−pheno = −0.324) compared to other network proteins (rank 9/175 among all proteins).”*

**(E) At the end of eq 12., after the s.t., should that “i=g,pr,m,ph” be “j=…”? Because there’s so “i” in the formula. In eq 13., it appears to be a “j”.**

*The typo has been corrected.*

**(F) Line 459, to me a correlation of 0.5 does not seem like it can be called “highly” correlated. Maybe moderately correlated. Maybe compare it to the distribution of correlation values within network and outside the network? Does that make it “high”?**

*We appreciate the reviewer’s suggestions and use “moderately correlated” instead. Additionally, we added more information to the paragraph to compare the correlation value to the distribution of the correlation value. For instance, when mentioning the correlation value between the molecular features, we specified that it is among their top 5 connections to other network molecular features based on Pearson’s correlation; when mentioning the correlation between molecular features and phenotype, we include their correlation rank when compared to other molecular features within the same molecular profile. Specifically, we mentioned the correlation between PCNA and tumor purity rank 1/175 of all proteins, and the correlation between KAT2A and tumor purity rank 9/175 of all proteins, below is the updated paragraph:*

***Section 0.14:*** *“We calculate the PageRank score for the SGTCCA-Net subnetwork and present the top 5 nodes for each molecular profile in Table 3. We observed that all of the molecular features in the table are significantly correlated with tumor purity. ACVRL1 and KAT2A are the only two nodes with low correlation to tumor purity (FDR > 0.001). To check whether these nodes contribute to the network, we examine the correlation between these two nodes and other molecular features of the subnetwork by looking at their top 5 connections (based on Pearson’s correlation) to other molecular features. We observed that ACVRL1 is moderately correlated with PCNA (ρbetween−omics = 0.474), which is highly correlated with tumor purity (ρomics−pheno = −0.564) compared to other network proteins (rank 1/175 among all proteins) and KAT2A (ρbetween−omics = 0.476), which is highly correlated with tumor purity (ρomics−pheno = −0.324) compared to other network proteins (rank 9/175 among all proteins).”*

**(G) It would be interesting to compare the features to cell type estimates from CIBERSORT (for example), which are already available for TCGA data. Or looking in single cell data for where certain genes are expressed, what cell types. Since the selected phenotype was tumor purity, the network is almost surely related to the quantity and type of cells other than tumor cells.**

*We followed up on the reviewer’s suggestion and checked the available cell type estimates data for TCGA breast cancer. The data output we found is from the following paper:*

*Craven, K. E., Gökmen-Polar, Y., & Badve, S. S. (2021). CIBERSORT analysis of TCGA and METABRIC identifies subgroups with better outcomes in triple negative breast cancer. Scientific Reports, 11(1), 4691.*

*When merging the cell type estimates with the final network output data, there are only 14 available samples, which doesn’t give enough statistical power to provide us with a reliable result. However, we did run CIBERSORT to estimate cell types and then performed the correlation analysis between cell type and network PC1:*

***Section 0.12.1:*** *……Furthermore, to identify connections between enrichment results and cell type compositions, we also use cell type estimates from TCGA breast cancer data to find the association between the final subnetwork and cell type composition using CIBERSORT [49]. CIBERSORT is a computational method that utilizes gene expression data to estimate the abundances of specific cell types within a mixed cell population, providing information on the cellular composition of complex tissues [50].* *Specifically, we take network PCs with significant associations to tumor purity and run Pearson's correlation between these PCs and the cell type estimates to identify which cell types are highly associated with the network.……*

***Section 0.14:*** *When running correlation analysis between network PC1 (only PC among the first 3 PCs with significant association to tumor purity) and cell type estimates from CIBERSORT, we found that the top 3 correlated cell types are T cells (CD8) (ρ = −0.781), Macrophages (M1) (follicular helper) (ρ = −0.528), and T cells (follicular helper) (ρ = −0.492). The strong correlations with cell types involved in immune responses align with the top enriched ontologies displayed in Figure 3a, such as lymphocyte activation and adaptive immune response, indicating that the network is capturing key aspects of the tumor’s immune landscape.*

*Additionally, we did the cell type analysis for COPDGene data:*

***Section 0.15:*** *……In particular, when checking the correlation between the network and cell type, we found that NetSHy PC1 is highly correlated with the percentage of neutrophil cells (ρ = -0.767), which may explain the significance of the neutrophil degranulation pathway…….*

**(H) Maybe table 4 could have an additional column, the proteins certainly need at least an ID or maybe a symbol.**

*We added ID for genes and proteins:*

**(I) With regard to figure 6, have a look at “biofabrics” as a way of visualizing network structures. Might find it useful in cases like this.**

*We appreciate the suggestion. We will have our comprehensive SGTCCA-Net pipeline built in an R package (release soon). In the package, when exploring the data visualization, we will consider incorporating “biofabrics”. Our current network can be visualized in different ways: (1) using Cytoscape software to communicate with R, which has more visualization flexibility, and (2) Shiny application from SmCCNet 2.0:* [*https://smccnet.shinyapps.io/smccnetnetwork/*](https://smccnet.shinyapps.io/smccnetnetwork/)*, which is a more convenient way to visualize the network but with less visualization flexibility. Our new package will also incorporate CIRCOS (*[*https://circos.ca/*](https://circos.ca/)*) environment to visualize the network.*

**(J) Figure 5b could use a legend to show how many and what colors are used for the cluster labels. It’s hard to see them as it is.**

*The updated figure 3(b) and 5(b) have included the color legend indicating the cluster ID.*

**(K) In Appendix S1, equation (1) provides an equation for covariance. In the second paragraph you write "Eq 1 calculates the higher-order correlation between vectors", but that's covariance, correct? Perhaps review the language around this across the manuscript? There seems to be some exchangeability between the terms that should be corrected. When deriving the correlation, I would expect to see cov(U,V) / sqrt( var(U) var(V) ) someplace, maybe I'm missing it.**

*We understand the reviewer’s concern on the language between covariance and correlation. In the original tensor canonical correlation analysis, when deriving the higher-order correlation, the authors use covariance and correlation interchangeably. For instance, in Section 4.2 of Luo et al., 2015, Equation 4.5 is not scaled by variance, but the author still calls it correlation. Technically, higher-order correlation doesn’t have an immediate extension as the traditional Pearson’s correlation. Therefore, like Sparse multiple canonical correlation analysis (SmCCA) (Hu et al., 2018), the objective function of Generalized Tensor Canonical Correlation Analysis is in fact maximizing the covariance. However, we understand the potential confusion caused by using terms interchangeably. Therefore, we adopt the naming convention suggested by the reviewer and uses higher-order covariance when defining our method since it is not scaled by variance, and we added some clarification that we use higher-order covariance as an unscaled measurement of higher-order correlation. Below is the clarification sentence we added:*

***Section 0.6.1:*** *In this work, the higher-order covariance defined above is the unscaled measurement of higher-order correlation since it is difficult to scale it to 0-1 scale mathematically by extending Pearson's correlation definition. Therefore, throughout the paper, we use the higher-order covariance value to represent the higher-order correlation.*

Minor:

⁃ Line 36, reference for SmCCA?

*The reference has been added.*

⁃ Line 152 “as follows:”

*It has been corrected.*

Reviewer #2: The manuscript by Liu et al. presents a novel framework for multi-omics network inference that addresses several limitations of existing methods. The proposed Sparse Generalized Tensor Canonical Correlation Analysis Network Inference (SGTCCA-Net) is an advancement of the previously developed Tensor Canonical Correlation Analysis (TCCA), incorporating higher-order correlations and several other improvements. SGTCCA-Net shows promise in effectively integrating multi-omics data, accounting for both higher-order and lower-order correlations and improving computational efficiency. The manuscript provides an important contribution, especially considering the rapid accumulation of multi-omics data. However, several points need to be addressed for clarity:

**1) It would be beneficial to include a figure illustrating the types of data SGTCCA-Net can handle, highlighting the dimensionalities of the data (e.g., genes vs. conditions/patients).**

*We appreciate the reviewer’s suggestion. In the workflow figure (Figure 2), it illustrates the types of data that SGTCCA-Net can handle. We realized that it doesn’t have the dimensionality in it. Therefore, we updated the figures to provide more insight into the dimensionality of the data and include some illustrating icons for molecular profiles (updated figure 2).*

*Additionally, we also include one paragraph illustrating the types of data our SGTCCA-Net pipeline can handle with dimensionality illustration:*

***Section 0.5:*** *Our pipeline is primarily designed for -omics data, such as transcriptomics, proteomics, metabolomics, and quantitative phenotypes associated with complex traits (see Figure 2). Additionally, it can handle other types of -omics data, such as ATAC-Seq and epigenetics data. All -omics datasets must include the same set of subjects (N) but can contain different numbers of molecular features, which may be either low or high dimensional.*

**2) The description for cases 1-3 in Experiments 0.11, as explained in the caption of Table 1, is crucial. It demonstrates the superiority of SGTCCA-Net in handling three types of data. This explanation should be included in the main text and presented in an orderly fashion. Additionally, the corresponding case names should be labeled in Table 1.**

*We appreciate the reviewer’s feedback on simulation strategies presentation. Table 1 is now annotated with Case 1-3. In addition, we include the caption information in the main text:*

***Section 0.11:*** *Under each latent factor simulation strategy, we proposed three distinct correlation structure setups (Case 1-3): Case 1 simulates all possible higher-order / lower-order phenotype-specific correlations (four-way, three-way, and pairwise correlations) as signal features; case 2 simulates only phenotype-specific four-way correlations as the signal features, and case 3 simulates all phenotype-specific three-way and pairwise correlation structures as the signal features. These signal correlation structures are simulated by latent factors mentioned above (red in Table 1). Furthermore, we also simulated non-phenotype-specific correlation structures to interfere with signal feature identification and test the robustness of each method (black in Table 1).*

**3) In Experiments 0.11, the evaluation metric, Area Under the Receiver Operating Characteristic Curve (AUC-ROC), is not well defined in the manuscript or the supplementary information. A clear definition and ROC plots should be included. The authors should also explain how this metric evaluates the performance of the methods.**

*We appreciate reviewer’s feedback on AUC score and its definition. Therefore, we’ve added the following content to define the AUC score, which is consistent with SmCCNet paper published in 2019:*

***Section 0.11:*** *Specifically, for each simulated feature, we identify and record the maximum edge value to other features in the adjacency matrix, treating this value as the predicted value. These predicted values are then scaled to fall within the 0-1 range. To evaluate the predictions, we compare them against the ground truth values, where each ground truth value is a binary indicator denoting whether a feature is a signal (1) or noise (0). The predictive performance is quantified using the Area Under the Receiver Operating Characteristic Curve (AUC-ROC). This involves sorting features by their predicted values, calculating the true positive rate (TPR) and the false positive rate (FPR) at various thresholds, plotting the ROC curve and computing the area under this curve. The AUC score effectively summarizes the model's ability to discriminate between signal and noise features, with a score of 1.0 indicating perfect discrimination and 0.5 indicating no discriminative ability (random guessing).*

*We measured AUC scores for each replication, and it is challenging to generate ROC curves for all cases and replications. Therefore, we have opted to display a table with median AUC scores and interquartile ranges, which is consistent with the methodology used in the SmCCNet paper published in 2019. This approach allows us to effectively summarize the performance of our methods across multiple replications, providing a clear and concise evaluation metric.*

**4) In Experiments 0.14, when using external software (e.g., MultiMiR), a brief description should be included to provide context.**

*We appreciate reviewer’s feedback. In section 0.14, we added one sentence to describe MultiMiR in general:*

***Section 0.14:*** *MultiMiR is an R package and database with a collection of microRNAs/targets from external resources, including validated microRNA-target databases and predicted microRNA-target databases, which was used to evaluate microRNA-target gene associations in the inferred networks [45].*

**5) The subheadings in the Results section currently refer to the dataset used, which is not particularly informative. The authors should consider using short sentences that describe the key take-home message of each section.**

*We appreciate the reviewer’s feedback and have changed all result subheadings to “Simulations Demonstrate Robustness and Accuracy of SGTCCA-Net”, “Relevant Biological Pathways Found for TCGA Breast Cancer Network Analysis Using SGTCCA-Net”, and “Relevant Biological Pathways Found for COPDGene Network Analysis Using SGTCCA-Net”.*

**6) The numbering of the subheadings needs to be revised. Remove the “0.” prefix and simplify the numbering system for clarity.**

*We appreciate the reviewer’s feedback. We used the template from journal to format the manuscript. All numbers will be removed to comply with the journal requirements later if the manuscript is accepted.*

Reviewer #3: The manuscript provides an insightful contribution to the field of multi-view dimension reduction. Below are some suggestions to enhance the clarity of the work:

**1) The manuscript would benefit from explaining why other dimension reduction methods were not listed in Introduction 02. Providing a rationale for the selection of the chosen methods, and discussing why others were not considered, would enhance the reader's understanding. Highlighting the unique or suitable aspects of these methods would be particularly beneficial.**

*We appreciate the reviewer’s comment on providing rationale for the selection of canonical correlation analysis. Therefore, we made some modifications on the section 0.2 to provide more background information about single-view dimensional reduction, then making transition to multi-view dimensional reduction, and state the drawbacks of other methods:*

***Section 0.2:*** *Dimension reduction is one of the more common goals of high dimensional data analysis. Many methods are proposed to achieve such a goal by projecting original data into the lower-dimensional embedding, which includes and is not limited to Principal Component Analysis (PCA) [18], t-Distributed Stochastic Neighbor Embedding (t-SNE) [19], and Uniform Manifold Approximation and Projection (UMAP) [20]. However, these methods are primarily designed for single-view datasets and do not inherently account for the complexity of multi-view data, in which multi-omics data is a particular example. In recent years, many multi-view dimensional reduction techniques have been proposed to address this limitation and effectively handle data with multiple views or modalities. For example, non-negative matrix factorization is implemented to cluster multiple data sets [21], and multi-view co-reduction is developed to preserve the locality within the view and the consistency between the views in the lower dimensional embedding of each view [22]. While these methods are proven to be effective for some multi-view tasks, they are not specifically designed to identify feature relationships and network inference across different views, which is the primary goal for multi-omics integration that uncovers the interaction between molecules with respect to trait of complex diseases. Another set of dimension reduction methods for multiple data sets are based on Canonical Correlation Analysis (CCA) [23], achieved this goal by finding the linear combination (canonical weight) that maximizes the correlation between two sets of data…..*

**2) Lines 26-30: When mentioning more general solutions, it would be helpful to clarify whether these solutions are specific to Canonical Correlation Analysis (CCA) or applicable to other methods as well.**

*We appreciate the reviewer’s feedback on canonical weight solutions. To clarify this, we added a few sentences for more description about the canonical weight solution and whether it is specific to canonical correlation analysis.*

*Section 0.2: ……In the context of CCA, there is typically more than one solution. These solutions collectively form a canonical weight matrix that is used to project each dataset into a lower-dimensional shared space. This projection approach is common in many multi-view methods for dimension reduction. The concept of using more than one solution to extract latent factors is shared by various multi-view dimension reduction techniques, each with its own specific methodology, but the extraction of multiple canonical weight solutions is specific to CCA……*

**3) Describing the symbols used in the formulas throughout the manuscript would aid reader comprehension.**

*A notations and symbols summary table are included in the supplementary material:*

**4) Additionally, ensure that GTCCA and other abbreviations are clearly defined in both the figure and the main text. Clear explanations of each part of the figures will help readers easily understand their meaning.**

*We appreciate the reviewer’s feedback and have defined all the abbreviations. The only seemingly abbreviated term, Turbo-SMT, according to the original publication, is not the abbreviation of any method. Additionally, we expand the caption of the workflow to help readers understand each part of the figure:*

**5) Lines 35-36: Providing a more detailed explanation of SmCCA would help readers understand this concept better.**

*We appreciate reviewer’s suggestions on adding detailed explanation of SmCCA, and make changes accordingly:*

***Section 0.2:*** *……* *A multiple canonical correlation method was developed to maximize the sum of pairwise canonical correlations [21]. Suppose that there are j = 1, . . . , K views, then the problem can be formulated as follows:*

*A penalty term can be added to the formulation above to achieve sparsity, which is called Sparse Multiple Canonical Correlation Analysis (SmCCA). Specifically, SmCCA introduces L1 penalty terms to the objective function, resulting in the following optimization problem:*

*Here, λ_j_ are tuning parameters controlling the level of sparsity for each view. The L_1_ penalty (||wj ||_1_) shrinks some elements of the weight vectors to be exactly zero to guarantee feature selection. This sparsity is particularly useful in high-dimensional settings, as it identifies a subset of features that are most relevant for the canonical correlations across multiple views. Therefore, SmCCA combines the benefits of multi-view analysis with feature selection, enhancing interpretability…..*

**6) Explaining the principles behind the algorithms/proposed method would help readers appreciate the rationale for using this proposed method. Including diagrams to illustrate the process of algorithms in the methods section could enhance understanding and accessibility.**

*We appreciate the reviewer’s suggestion on adding conceptual diagrams and illustrations to guide readers through complete process. We have included a conceptual diagram (new Figure 2) to illustrate the process, with more description to guide readers through the rationale behind our method.*

***Section 0.5:*** *Figure 2 illustrates the conceptual framework of the SGTCCA-Net algorithm. The process begins with the implementation of Sparse Generalized Tensor Canonical Correlation Analysis (SGTCCA) to perform feature selection, targeting higher and lower-order correlation structures of interest. The next step involves network analysis to identify interactions between molecular features across different molecular profiles, leading to the construction of global networks based on the SGTCCA results. However, the global network may include molecular features that are less related to the phenotype of interest. To address this, a network pruning algorithm is applied to remove these irrelevant nodes. Following pruning, the resultant subnetwork may still contain weaker edges connecting less correlated molecular features. To refine the network further, an edge filtering step based on Pearson’s correlation is performed, eliminating weaker edges and enhancing the network’s overall robustness.*

**7) Line 134: Clarifying what is meant by the directionality (positive/negative) of higher-order correlation and why it is not interpretable would provide valuable insight.**

*We appreciate the suggestions and have included one paragraph in the discussion section explaining that directionality is the drawback of the higher-order correlation. However, post-hoc analysis can be performed afterwards to identify pairwise correlations between molecular features:*

***Discussion:*** *“One potential drawback of higher-order correlation is that it measures the simultaneous relationship among multiple features, making it difficult to determine the directionality of effects through mathematical formulation alone. However, a post-hoc analysis can be conducted on the higher-order correlation results to examine the direction of effect for each pair of features. For example, after identifying the 4-way correlation between a gene, a protein, a metabolite, and a phenotype, a post-hoc analysis can be performed to look at the pairwise Pearson's correlation between each pair of features, such as the gene and protein. This approach allows researchers to investigate the directionality of relationships between individual pairs of features, providing a clearer understanding of the underlying biological interactions.”*

*Additionally, in our experiments, we have included paragraphs that examined the pairwise association between molecular features using Pearson’s correlation. For example, in TCGA breast cancer example, we provided the following example:*

***Section 0.14:*** *“We calculate the PageRank score for the SGTCCA-Net subnetwork and present the top 5 nodes for each molecular profile in Table 3. We observed that all of the molecular features in the table are significantly correlated with tumor purity. ACVRL1 and KAT2A are the only two nodes with low correlation to tumor purity (FDR > 0.001). To check whether these nodes contribute to the network, we examine the correlation between these two nodes and other molecular features of the subnetwork by looking at their top 5 connections (based on Pearson’s correlation) to other molecular features. We observed that ACVRL1 is moderately correlated with PCNA (ρbetween−omics = 0.474), which is highly correlated with tumor purity (ρomics−pheno = −0.564) compared to other network proteins (rank 1/175 among all proteins) and KAT2A (ρbetween−omics = 0.476), which is highly correlated with tumor purity (ρomics−pheno = −0.324) compared to other network proteins (rank 9/175 among all proteins).”*

**8) Ensuring consistency in the row sizes of the data shown in A1 and A1 in S3 text.**

*The typo on row sizes has been fixed.*

**9) Providing citations to support Theorem 1, if it has been previously validated, would ensure it has a strong mathematical foundation.**

*We appreciate the reviewer’s feedback on Theorem 1. The proof of this theorem is the direct extension of a previously established proof for tensor decomposition equivalency. We added one sentence before stating the Theorem 1: “(proof in S2 Text and is the direct extension of proof of tensor decomposition equivalency [32])”*

**10) Clarifying why DIABLO was specifically chosen for comparison and highlighting its relevance and significance would improve the comparison discussion. Additionally, discussing other methods that could be used for comparison alongside SmCCNet and DIABLO would provide a more comprehensive evaluation of the proposed approach.**

*We appreciate the reviewer’s suggestions on explaining why we compare SGTCCA-Net with SmCCNet and DIABLO and added a clarification at the experiment section.*

***Section 0.11:*** *SmCCNet and DIABLO are chosen because they are the only two methods that consider molecular interactions with respect to phenotype using a network approach. Both methods utilize a similar canonical correlation-based technique to generate the interaction network. By comparing SGTCCA-Net with these two methods, we can observe the direct improvements of SGTCCA-Net, as it incorporates higher-order correlations in the analysis.*

**11) Including a thorough discussion of how the proposed method leads to the results and an analysis of whether the results from the data are reasonable. Discussing the methodology's effectiveness and the reasonableness of the results obtained would provide valuable context and insight.**

*We appreciate reviewer’s feedback.*

*Regarding how the proposed method leads to the results, the stepwise rationale was explained in method section 0.5. Additionally, to re-iterate and summarize this, we added the following paragraph to the discussion section:*

***Discussion:*** *……Our proposed pipeline simplifies the process for users by requiring only the specification of correlation structures of interest, typically related to traits of complex diseases. Based on these defined correlation structures, the SGTCCA algorithm identifies molecular features that are involved in one or more of these relationships. Following this, the network analysis step reconstructs the interaction networks in a 2D space, enabling the visualization and interpretation of the internal biological mechanisms that link molecular features to the disease. This approach provides a clear and comprehensive understanding of the molecular interactions driving complex diseases, making the results more interpretable and actionable for researchers……*

*Regarding the analysis of whether the results from the data are reasonable, in the experiment section (section 0.13 and 0.14), all the interpretation revolves around whether the results from data are reasonable. We validated this through various angles, which includes and not limited to finding the univariate correlation between network molecular features and phenotype; finding the NetSHy summarization score correlation to phenotype; conducting enrichment analysis to identify relevant pathways; substantiate the weaker network nodes are still important in the networks, and more. Additionally, to avoid confusion, we added the following paragraph in the discussion section to summarize all the interpretation:*

***Discussion:*** *……Our proposed method demonstrated its effectiveness by successfully capturing both higher-order and lower-order correlations within multi-omics data, leading to the construction of robust multi-omics networks. The results obtained from both simulation studies indicate that SGTCCA-Net effectively identifies phenotype-specific molecular interactions even in the presence of noisy data. The higher AUC scores observed across various scenarios confirm the reasonableness of the results, as SGTCCA-Net consistently outperformed other comparable methods SmCCNet and DIABLO. These findings validate the ability of the methodology to uncover complex molecular relationships. Additionally, we applied SGTCCA-Net to both COPDGene and TCGA breast cancer datasets, which demonstrates its robustness and practical utility in real-world scenarios. In the TCGA breast cancer data, SGTCCA-Net successfully identified phenotype-specific networks related to tumor purity, demonstrating superior performance compared to SmCCNet, with a higher NetSHy network summarization score and stronger correlations between the molecular features and tumor purity. These results indicate the method’s ability to capture intricate multi-omics relationships that are critical for understanding cancer biology. Similarly, in the COPDGene dataset, SGTCCA-Net effectively uncovered networks associated with pulmonary function, revealing key molecular interactions that contribute to chronic obstructive pulmonary disease (COPD). Interestingly, for both studies, the biomarkers identified are not limited to those with strong phenotype correlations; we also identified molecular features with weak phenotype correlations that are highly connected to other nodes and contribute to the network. Furthermore, we validated our findings using various techniques, including enrichment analysis of biological pathways and miRNA-target relationships. These validations confirm the enhanced performance of SGTCCA-Net over SmCCNet and the ability to find relevant pathways. Furthermore, we also demonstrate the top higher-order correlation associated with the final subnetwork. These applications demonstrate that SGTCCA-Net not only produces reasonable and reliable results but also offers meaningful insights into complex diseases, making it a valuable tool for multi-omics network inference in diverse biomedical research contexts……*

Best regards

Weixuan Liu (on behalf of all co-authors)
