# Supplementary material for: A generalized higher-order correlation analysis framework for multi-omics network inference
Source: PLoS Comput Biol. 2025 Apr 14;21(4):e1011842. doi: 10.1371/journal.pcbi.1011842 (PMC11996223; doi:10.1371/journal.pcbi.1011842)
Supplement: Attachment — Submitted filename: Second-Response-Letter-KK.docx [file pcbi.1011842.s003.docx]

Dear Editor,

Thank you so much for the quality assessment on our manuscript “A Generalized Higher-order Correlation Analysis Framework for Multi-Omics Network Inference”. We have carefully revised it based on your feedback and have made corresponding changes. Below, we respond to each point:

Best regards,

Weixuan Liu (on behalf of all co-authors)

Review comments:

*1. line 176-177 You mention that the original TCCA faces challenges in interpreting the directionality of higher-order correlations. However, the proposed SGTCCA-Net seems to share a similar limitation regarding the interpretability of directionality. Could you explain what has been improved in SGTCCA-Net compared to the original TCCA regarding this limitation?*

We appreciate reviewers feedback on the directionality of higher-order correlation. We adjusted the description here to avoid confusion. The new paragraph indicates that the challenge is that the sign of the higher-order correlation cannot be interpreted as the direction of an effect. Additionally, we mentioned that even though the higher-order correlation in TCCA is not interpretable, the directionality can still be inferred through the subsequent network analysis step. Below is our updated paragraph:

Section 0.6.1: … The original TCCA algorithm has two major issues: (1) while the magnitude of the higher-order correlation is informative, the sign (positive or negative) of the higher-order correlation is not interpretable for the direction of a relationship, especially when there are more than three datasets, and (2) the correlation effect is canceled when there is an odd number of datasets (see Appendix S1 for details). To address these limitations, we proposed a new tensor canonical correlation analysis formulation that (1) avoids the negativity of the higher-order correlation value and (2) calculates the higher-order correlation value for an odd number of views with slight adjustments. Although the directionality of the higher-order correlation cannot be directly inferred, the directionality of the interaction between any two features can be inferred during the network analysis step described later….

*2. In Eq(5), show how covariance is calculated for an odd number of views in the main text would help highlight what sets your method apart from the original TCCA.*

We moved the covariance calculation from supplement to the main text in section 0.6.1:

 *3. Each step in Figure 3 needs a bit more explanation. It would be easier to understand if the figure’s process is clearly described. Also, linking this figure with Algorithms 1 and 2 would help readers follow along better.*

We expanded the description of Figure 3:

Workflow of SGTCCA-Net pipeline for multi-omics network inference. The workflow consists of four example input data: transcriptomics (gene), proteomics (protein), metabolomics (metabolite), and phenotype, each with $N$ subjects. Transcriptomics, proteomics, and metabolomics data have $d_1$, $d_2$, and $d_3$ features, respectively, and phenotype data has one feature.} The pipeline first calculates the covariance density of each molecular feature, then the algorithm randomly selects a subset of the molecular features, biased towards features with high covariance density (Algorithm 1 in S3 Text, molecular features with high covariance density more likely to be selected). Based on the selected features, Generalized Tensor Canonical Correlation Analysis (GTCCA, Algorithm 2 is run to identify molecular features involved in higher/lower-order correlation of interest. Based on the GTCCA result, an affinity matrix between molecular features is constructed to identify the interaction between selected molecular features (Algorithm 2 in S5 Text). Network pruning then further filters out weaker molecular features and edges in the affinity matrix(Algorithm 2 in S5 Text).

*4. line 195, z_1,2,3=z1z2z3, Could you clarify why they are being grouped, what is the relationship between these variables?*

We expanded the section with more context and examples:

Section 0.6.: As mentioned above, the higher-order relationship may not imply multiple lower-order or pairwise relationships, and vice versa. Below, we provide some scenarios as an illustration. Suppose there are 4 different feature vectors z1, z2, z3, z4 ∈ R N×1 that can be constructed by either (1) letting z1, z2, z3 be less correlated with each other but all strongly correlated with z4 (e.g., this can be achieved when the correlation structure is z4 = z1 + z2 + z3), or (2) letting z1, z2, z3 be highly correlated with each other but independent of z4. According to Equation 5, the 4-way higher-order covariance value between them can be regrouped to calculate the element-wise multiplication of the first three vectors, then element-wise multiplied by the last vector, which can be reformulated as: ρ(z1, z2, z3, z4) = | 1 n (z1z2z3z4) T 1| = | 1 n (z1,2,3z4) T 1|, (6) where z1,2,3 = z1z2z3 is the element-wise multiplication of z1, z2 and z3. In one particular scenario, a strong 4-way correlation between z1, z2, z3, z4 can be constructed by letting z1, z2, z3 be independent of each other, and z4 be z3 plus a small random noise. In this case, the 4-way correlation between z1, z2, z3, z4 does not imply the 3-way correlation between z1, z2, z3; In another particular scenario, a strong 3-way correlation between z1, z2, z3 can be constructed by letting z1, z2, z3 be strongly correlated with each other, but if z4 is independent of z1,2,3, then the 4-way correlation does not exist between z1, z2, z3, z4

*5. There are several numerical expressions throughout the paper displayed as fractions (/) or as pairs (/,*) without sufficient explanation. If these numbers are not actual fractions, please consider rewriting them in a more appropriate format. Additionally, clarify whether these correspond to the definition of the minimally/maximally sized final subnetwork. For instance, in line 661, the meaning of (18:0/18:1) needs further elaboration to avoid confusion.*

The ‘:’ and “/” notation are not fractions but the naming convention used for metabolite names that should not be altered. See <https://www.metabolomicsworkbench.org/databases/refmet/refmet_help.php> and https://www.metabolon.com/blog/lipid-nomenclature-data-table/ for more information.

*6. The comparison with SmCCNet mentions that it selects fewer validated/predicted target features and that the final network size is smaller. While this could suggest that selecting more features is better, it’s important to note that in sparse problems, quality is often prioritized over quantity. Some methods intentionally select fewer, more relevant features.*

We appreciate the reviewer’s suggestion and have included more information on why in the context of finding the connection between miRNA and genes, the SGTCCA-Net network can extract not only more genes but also those of higher quality (based on percentage supported byevidence in multiple sources). Below is the updated paragraph:

Section 0.14: We ran MultiMiR on the miRNAs from each subnetwork to evaluate the quality and relevance of miRNA-gene connections. For the SGTCCA-Net network, among 1288 global network genes with non-zero canonical weight, 179/697 (13.9%/54.1%) are validated/predicted target genes of miRNAs in the subnetwork. These targets correspond to 202/1590 unique miRNA-gene connections, considering that each gene can be targeted by multiple miRNAs. Further quality assessment revealed that 75/1033 (37.1%/65.9%) validated/predicted miRNA-gene connections are supported by more than one database, indicating robust evidence for these interactions. In comparison, for the SmCCNet network, among 1024 global network genes with non-zero canonical weight, only 102/212 (10.0%/20.7%) are validated/predicted target genes of miRNAs in the subnetwork. This results in 81/286 unique miRNA-gene connections, with only 35/134 (43.2%/46.9) supported by more than one database. While SmCCNet identifies fewer miRNA-gene connections, the lower proportion of validated or consensus connections suggests a more conservative feature selection approach, potentially prioritizing sparsity over coverage. This result indicates that SGTCCA-Net identifies a greater number of miRNA-gene connections, many of which share consensus across multiple databases, suggesting higher confidence and relevance. Enrichment analysis of the 179 validated targets in SGTCCA-Net subnetworks (S2 Fig) reveals similar ontology categories to the gene/protein enrichment analysis, including lymphocyte differentiation and the adaptive immune system. These findings highlight SGTCCA-Net’s ability to uncover biologically meaningful and well-supported connections while balancing quality and coverage.

*7. It would help readers if you explain what would be considered "good" values or ranges for the test results you present. Providing context will make it easier to interpret the results.*

We appreciate reviewer’s feedback and have included information about how network correlation compares to the range possible by individual features only for the two datat sets (TCGA and COPDGene).

Section (0.14) (TCGA): After running **the** network analysis pipeline with SGTCCA and SmCCNet for the phenotype of tumor purity, the final network size for both SGTCCA-Net and SmCCNet is 300. Specifically, there are 128 genes, 125 miRNAs, and 47 proteins in the SGTCCA network, while there are 128 genes, 59 miRNAs, and 113 proteins in the SmCCNet network. The NetSHy network summarization score correlation with respect to tumor purity is 0.689 and 0.500 for SGTCCA-Net and SmCCNet respectively. These are relatively large values as the maximum and 90^th^ quantile of the univariate correlation between any of the molecular features and tumor purity are 0.76 and 0.45 respectively

Section 0.15 (COPD): After running the network analysis pipeline with SGTCCA-Net and SmCCNet for the FEV1 phenotype, the final network size for SGTCCA-Net is 170, while SmCCNet has a final network size of 117. The NetSHy summarization score for SGTCCA-Net correlation with respect to FEV1 is 0.211, while for SmCCNet it is only 0.162. These are relatively large values as the maximum and 90^th^ quantile of the univariate correlation between any of the molecular features and FEV1 are 0.28 and 0.12 respectively
